# Pharmacists and family physicians: improving interprofessional collaboration through joint understanding of our competencies

**DOI:** 10.3389/fphar.2013.00151

**Published:** 2013-12-05

**Authors:** Katherine Stringer, Vernon Curran, Shabnam Asghari

**Affiliations:** ^1^Discipline of Family Medicine, Faculty of Medicine, Memorial University of NewfoundlandSt. John's, NL, Canada; ^2^Faculty of Medicine, Memorial University of NewfoundlandSt. John's, NL, Canada; ^3^Primary Health Care Research Unit, Faculty of Medicine, Memorial University of NewfoundlandSt. John's, NL, Canada

**Keywords:** interprofessional collaboration, interprofessional education, competency based education, multidisciplinary teams, medical education, interdisciplinary communication

Interprofessional collaboration (IPC) is an integral part of the practice of Medicine and Family Medicine. The World Health Organization (WHO) defines IPC as occurring when “multiple health workers from different professional backgrounds work together with patients, families, carers and communities to deliver the highest quality of care” (WHO, 2010). To provide effective, patient-centered care, family physicians must collaborate with other health and social care providers. This is especially true in Canada where there is an increasing level of chronic care and multimorbidity. Between 1998/99 and 2008/09 the prevalence of diagnosed diabetes among Canadians has increased by 70%. Over 36.5% of Canadian adults with diabetes report two or more other serious chronic conditions (hypertension, heart disease, chronic obstructive pulmonary disease, mood disorder, and/or arthritis) in addition to diabetes, and 12.5% report having three or more (Frank, 2005). The Collaborator role has therefore appropriately been included in the CanMEDS framework of competencies by the College of Physicians and Surgeons of Canada (Frank, 2005) and the College of Family Physicians of Canada (CFPC) (Tannenbaum et al., 2009). These frameworks are used in the design and accreditation of undergraduate and family medicine curricula as well as to improve patient care by ensuring that training programs in family medicine are responsive to societal needs (Tannenbaum et al., 2009).

The delivery of responsive, effective, and high-quality patient care is indeed a complex activity. It demands health care professionals collaborate in an effective manner (Reeves et al., 2013). IPC can be challenging and barriers such as role identification and clarification of expectations continue to be experienced in practice (Lapkin et al., 2013). Interprofessional education (IPE) offers a possible way to improve IPC and patient care and now forms an essential part of medical school curricula in many countries including Canada, USA, UK, and Australia (Reeves et al., 2013). IPE is described as occurring when two or more professions learn about, from and with each other to enable effective collaboration and improve health outcomes (WHO, 2010). A fundamental premise of IPE is that if health professional students learn together they will be better prepared for IPC and teamwork. IPE is a therefore considered a necessary step in preparing a “collaborative practice-ready” workforce that is better prepared to respond to local health needs. A collaborative practice-ready health provider is someone who has learned how to work in an interprofessional team and is competent to do so (WHO, 2010). The duration of the positive effects of IPE programs for medical and health care students, however, and their transferability to clinical practice is still unclear.Recent meta-analyses report inconclusive evidence on the effectiveness of IPE on outcomes such as collaborative professional practice or patient care (Lapkin et al., 2013; Reeves et al., 2013). In order to understand what needs to be improved upon in IPE, a deeper understanding of the essential components of a successful collaborative relationship is required (WHO, 2010).

Competence, or the knowledge and skills base underlying a particular profession, can also be considered a profession's “cognitive map.” Good teamwork relies on a joint understanding of one's own as well as other team members' cognitive map (Drinka and Clark, 2000). In clinical practice this requires that a profession not only clearly describe their own roles and responsibilities to other professionals but also have an awareness of other professions' competencies in relation to their own (Drinka and Clark, 2000); This is a key competency that has been linked with improving team communication, coordination of care and patient safety and is included in the CanMEDS description of a collaborator (Frank, 2005; CMPA, 2007; Prada et al., 2007; Frank and Brien, 2008).

Defining one's own professional competencies is therefore an important first step in effective IPC. One approach to gathering the necessary information to determine what competencies are required for a particular profession is to ask those already active and skilled in the profession itself. This approach was used by the CFPC in the development of the competency–based objectives in family medicine using practicing family physicians as their source of information (Allen et al., 2011). This is now the standard used to define competence for the purpose of certification in family medicine by the CFPC.

Another approach includes consultation with others. Realizing the importance of patients, physicians, and other health care professionals in the delivery of health care, research in the 1990's by the Ontario provincial government in Canada sought both public and other health care professionals input in an attempt to define the expectations of physicians performance. The results showed significant difference of opinion between physicians and other health care professionals on items referring to distribution of responsibility, control, and authority of physicians within the health care system at that time (Neufeld et al., 1998). Recently, research on the perceived roles of internal medicine postgraduate trainees (self and other health care professionals) at patient discharge, illustrates a similar lack of consensus (Card et al., 2013).

Pharmacists represent the one health care profession all family physicians are most likely to collaborate with in providing optimal patient care. A recent UK study on the collaborative relationship between community pharmacy and general medicine, highlighted the dynamic nature of the relationship and the key components of collaboration. These included the importance of trust, communication, professional respect, and “knowing each other” when referring to roles, abilities and responsibilities (Bradley et al., 2012). Just as the medical profession has it's own set of defined competencies, so does pharmacy. The National Association of Pharmacy Regulatory Association includes professional collaboration and teamwork in its professional competencies for Canadian pharmacists (NAPRA, 2007). As part of the recent extension of prescribing rights to pharmacists in Canada, there has been extensive research conducted around family physicians' knowledge, perceptions and expectations of pharmacists' competencies and how this impacts the collaborative relationship (Farris, 2005; Bryan et al., 2009; Farrell et al., 2010; Dey et al., 2011). To date, however, there has been little work undertaken on pharmacists' or other professions' knowledge, perceptions or expectations of family physician competencies and how this may inform the collaborative relationship to further develop IPE curricula and ensure better patient care.

Our research team is exploring pharmacist's expectations of the competent family physician using an adaptation of the CFPC's original competency questionnaire distributed to practicing family physicians (Allen et al., 2011). An iterative approach using the Delphi method will seek to examine the perspectives of pharmacists on family physicians' competencies. A series of Delphi questionnaires will be administered to a panel of experts including rural, urban, academic, and non-academic collaborating pharmacists. Questions will focus on disease states, decision-making, judgment, and clinical situations requiring collaboration. The resultant definition of family medicine competencies from the viewpoint of collaborating pharmacists will then be compared to that defined by the CFPC. Commonalities and discrepancies will be analyzed to identify gaps and develop IPE methods to enhance collaborative competence in family medicine and pharmacy trainees.

Continued research in this area is essential to deepen our understanding of the essential components of the interprofessional relationship. Understanding and clarifying role expectations is central to this and can provide valuable information when designing and reviewing professional competency frameworks and IPE curricula. As more research becomes available, the effectiveness of these programs on outcomes such as IPC in clinical practice and patient care should be reviewed.
